# Human Electroretinography Shows Little Polarity Specificity Following Full-Field Ramp Adaptation

**DOI:** 10.1167/iovs.65.10.11

**Published:** 2024-08-06

**Authors:** Shalila T. Freitag, Maren-Christina Lengle, Sascha Klee, Sven P. Heinrich

**Affiliations:** 1 Eye Center, Medical Center—University of Freiburg, Freiburg, Germany; 2 Faculty of Medicine, University of Freiburg, Freiburg, Germany; 3 Institute of Biomedical Engineering and Informatics, Ilmenau University of Technology, Ilmenau, Germany; 4 Department of General Health Studies, Division Biostatistics and Data Science, Karl Landsteiner University of Health Sciences, Krems, Austria

**Keywords:** ramp aftereffect, dynamic adaptation, brightness perception, human electroretinogram

## Abstract

**Purpose:**

The ramp aftereffect, a visual phenomenon in which perception of light changes dynamically after exposure to sawtooth-modulated light, was first described in 1967. Despite decades of psychophysical research, location and mechanisms of its generation remain unknown. In this study, we investigated a potential retinal contribution to effect formation with specific emphasis on on-/off-pathway involvement.

**Methods:**

A 100 ms flash electroretinogram (ERG) was employed to probe the adaptive state of retinal neurons after presentation of stimuli that were homogenous in space but modulated in time following a sawtooth pattern (upward or downward ramps at 2 Hz). Additionally, a psychophysical nulling experiment was performed.

**Results:**

Psychophysics data confirmed previous findings that the ramp aftereffect opposes the adapting stimuli in ramp direction and is stronger after upward ramps. The ERG study revealed significant changes of activity in every response component in the low-frequency range (a-wave, b-wave, on-PhNR, d-wave and off-PhNR) and high-frequency range (oscillatory potentials) in amplitudes, peak times, or both. The changes are neither specific to the on- or off-response nor antagonistic between ramp directions. With downward ramp adaptation, effects were stronger. Neither amplitudes nor peak times were correlated with perception strength. Amplitudes and peak times were uncorrelated, and the effect diminished over time, ceasing almost completely with three seconds.

**Conclusions:**

Despite abundant effects on retinal responses, the pattern of adaptational effects was not specific to the sawtooth nature of adaptation. Although not ruling out retinal contributions the present findings favor post-retinal mechanisms as the primary locus of the ramp aftereffect.

In 1967, Anstis^1^ first described the ramp aftereffect, a visual phenomenon in which human perception is altered by adaptation to a sawtooth stimulus. When a luminance change following a sawtooth function is presented, a subsequent static stimulus will be perceived as changing its luminance dynamically. The direction of the perceived change is dependent on the polarity of the sawtooth. For upward ramps (i.e., slow rising phases and interposed rapid drops of luminance) the ramp aftereffect will be one of subjective dimming, whereas after downward ramps (i.e., slow decreases of luminance followed by rapid jumps to high luminance) a brightening is perceived. Sawtooth adaptation can also induce motion perception if followed by a test patch with luminance gradation.^1^ The underlying physiological mechanisms are still unknown, although psychophysical investigations have provided key insights into the ramp aftereffect's governing principles. For instance, temporal frequency does not seem to influence effect strength,^2^ and the ramp aftereffect does not result from adaptation to contrast but purely to light.^3^ Experiments with artificial pupils have excluded pupillary dilation as its possible source.^1^ The large spatial coarseness of the ramp aftereffect, around four to five times larger than the receptive fields of retinal ganglion cells (RGCs),^4^ suggests a cortical origin. Furthermore, an electroretinogram (ERG) study on another type of directional dynamic adaptation, namely motion adaptation, has not found evidence of an involvement of the retina.^5^ In contrast, the lack of interocular transfer narrows down possible sites of ramp adaptation to those that process visual information monocularly.^1^ Anstis and Harris^4^ proposed the retina but also the lateral geniculate nucleus or the midbrain to represent viable places of origin but excluded the V1 on account of incongruent spatial grain between receptive fields and ramp aftereffect. Comparing ramp aftereffect strength between polarities shows an asymmetry. The ramp aftereffect is stronger after adaptation to upward ramps.^2^ This may hint toward two discrete pathways like the transient on- and off-pathways as suggested by Anstis and Harris.^4^ On- and off-pathway signal luminance increments and decrements and originate in the bipolar cells (BCs) of the retina.^6^^,^^7^ Following roughly the push-pull model first proposed in cats,^8^ light-on events lead to an activation of on-type BCs, which have excitatory connections to on-RGCs while inhibiting off-RGCs directly or indirectly via amacrine cells.^7^^,^^9^^–^^11^ After light-off events, off-BCs activate off-RGCs and inhibit on-RGCs, again with or without recruitment of amacrine cells.^7^^,^^9^^,^^11^ Two aspects are particularly crucial. First, as implied by but not limited to the push-pull model of RGC activation, the two pathways do not relay information in isolation but do indeed interact and influence each other. Second, their anatomy, functionality, and crosstalk are asymmetric in nature (e.g., bigger receptive field sizes and faster responses in the on-pathway, as well as more inhibition from on- to off-pathway than vice versa).^12^^–^^15^ As Anstis and Harris^4^ argued already, it might be this asymmetry that allows the formation of the ramp aftereffect, instead of equal responses in mirrored pathways cancelling each other out.

Using long flashes to elicit a flash electroretinogram (fERG) allows for the separation and comparison of on- and off-pathway responses in the retina,^16^ potentially revealing the selective effect of sawtooth polarity. A plethora of cell types contributes to the fERG, with the most prominent components being the low-frequency high-amplitude components. In the response to light-on the fERG shows the a-wave (photoreceptor activity),^17^^–^^22^ the b-wave (antagonistically: on- and off-BCs)^23^^–^^25^ and on-photopic negative response (PhNR) (RGC and amacrine cell activity).^26^^,^^27^ In the response to light-off there are the d-wave (interplay of on- and off-BCs plus receptor activity)^25^^,^^28^ and the off-PhNR (RGCs and amacrine cells).^27^^,^^29^ More response components, the so-called *oscillatory potentials* (OPs), can be isolated by applying a high-pass filter at around 75 Hz.^30^ Their origin is poorly understood, although they are hypothesized to reflect a feedback loop relaying information from proximal to distal parts of the retina.^31^^–^^33^ OPs have also been suggested to contribute to adaptational processes in the inner retina.^34^ This makes them candidates as contributors to ramp aftereffect formation despite their elusive origins.

So far, many psychophysical studies have provided important contributions to characterizing the ramp aftereffect. However, to our knowledge, an electrophysiological assessment of neuronal correlates of the hypothesized retinal changes has not been conducted yet. The present fERG study aims at filling this void by probing the adaptive state of retinal cells after exposure to sawtooth modulated light.

## Material and Methods

### Participants

Data was obtained from 20 consenting informed adults between the ages of 20 and 38 (median: 24 years; 13 female and 7 male) with no known ophthalmological, psychiatric or neurological disorders. All had normal or corrected-to-normal visual acuity (decimal acuity ≥1.0) as confirmed with the Freiburg Acuity and Contrast Test (FrACT).^35^ Psychophysical and electrophysiological experiments were conducted in the same participants on the same day with the ERG being recorded after the nulling task. Total duration per participant was around three to three and a half hours. Neither refractive correction nor mydriasis was applied during measurements. The study followed the tenets of the Declaration of Helsinki and was part of a project approved by the local institutional review board.

### Visual Stimulation

**Figure 1.**
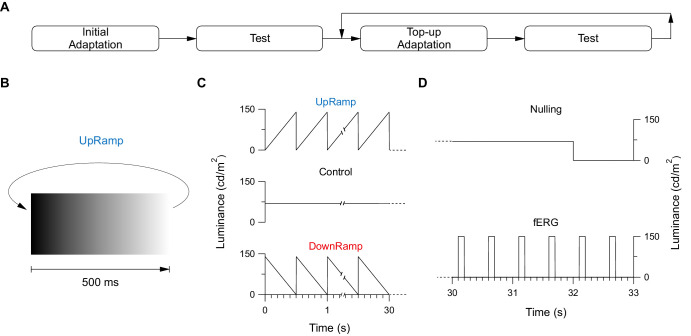
Visual stimulation for each experiment and condition. (**A**) Overview over the general sequence of adaptation and test phases in each experiment. After a first “initial adaptation” the aftereffect was probed in a “Test” phase, then topped up via a “top-up adaptation” and tested again. Topping up and testing were repeated several times. The adaptation differed between conditions, “UpRamp,” “DownRamp,” and “Control,” as shown in **C**. Initial and top-up adaptation could differ in duration. The test stimuli differed between the psychophysical Nulling and the electrophysiological fERG experiment, both shown in **D**. (**B**) One period (500 ms) of adapting stimulus showing the gradual change in luminance exemplified by the UpRamp condition. Starting with light off (0 cd/m^2^, *black*), the luminance gradually increased until it reached the maximum luminance (140 cd/m^2^, *white*). In a cyclic sequence (UpRamp in **C**), luminance fell back to light-off, gradually brightened again, fell back to light-off, etc. (**C**) Overview of adaptation luminance time courses for all three conditions: UpRamp with ramps of increasing light (*top graph*; single period also shown in **B**), DownRamp with gradually dimming ramps (*bottom graph*) and Control, a constant light at mean luminance (70 cd/m^2^; *middle graph*). The adaptation stimulus was repeated at 2 Hz for 30 seconds (in the nulling experiment, the top-up adaptation was shown for 10 seconds). All three conditions were tested in both experiments and followed by one of the test stimuli sketched in **D**. (**D**) Test stimuli as used in the psychophysical nulling (*top graph*) and the ERG (*bottom*) experiment as luminance over time. During the nulling, aftereffect perception was tested by presenting a constant luminance (70 cd/m^2^) for two seconds in the first trial and for one second with an individually adjusted ramp slope in top-up trials before light-off. In the ERG experiment responses were elicited by six flashes (150 cd/m^2^, 100 ms) presented at 2 Hz.

All measurements were taken binocularly using a Ganzfeld bowl (Ganzfeld Q450 stimulator; Roland Consult Stasche & Finger GmbH, Brandenburg an der Havel, Germany) with the stimuli produced by a custom-installed white-light (3000 K) LED panel (Queen.Y COB Chip, Amazon ASIN B08J84FX6R). The LED array was controlled by a purpose-written software running on a single-board computer (Raspberry Pi 4, Model B; Raspberry Pi Trading Ltd, Cambridge, United Kingdom). Light levels were controlled through pulse-width modulation of a carrier signal with a frequency of 100 Hz for the adapting stimuli and 250 Hz for the flashes used to elicit an ERG. A black dot at the back of the Ganzfeld bowl served as a fixation target. Identical adapting stimuli, “UpRamp,” “DownRamp,” and “Control,” were used in both psychophysical and electrophysiological experiments. In the UpRamp condition sawtooth-modulated luminance stimuli with upward-going ramp phases and rapid drops to black where presented, whereas DownRamp used adapting stimuli with downward-going ramps and rapid jumps to maximal brightness (Figs. 1B, 1C). Both were presented at a frequency of 2 Hz and a maximal luminance of 140 cd/m^2^. Static light at 70 cd/m^2^, equal to the mean luminance of the sawtooth ramps, was used in the control condition. All light intensities were measured with the Luminance Meter LS-100 (Konica Minolta, Inc., Tokyo, Japan). Duration of adaptation varied between experiments. Each adaptation phase was followed by a test phase during which either the participant judged and reported their percept (psychophysical nulling experiment) or fERGs were recorded (ERG experiment); thereafter the adapted state was restored and testing was repeated multiple times (Fig. 1A). An adaptation phase and the following test phase together constituted one trial. A fixed number of trials made up a run.

#### Experiment 1: Nulling

The preliminary nulling experiment aimed at assessing the perception of the ramp aftereffect and was modeled after a study by Arnold and Anstis.^2^ During the test phase after each adaptation phase, a physically counteracting probe stimulus was presented and, in response to the participant's reported percept of the ramp aftereffect, the subsequent probe stimulus was adjusted in strength. Going through several iterations, this allows for finding the probe stimulus strength that compensates (“nulls”) the ramp aftereffect. This general concept was implemented as follows.

Starting the first trial of every run, participants viewed an adapting stimulus (UpRamp, DownRamp or Control) for 30 seconds. In a pilot study, ramp aftereffect duration had typically reached its maximum after 20 seconds of adaptation, exceptionally after 30 seconds. Hence, the aftereffect duration should be maximal after initial adaptation. A short beep indicated the beginning of the subsequent test phase. During the test phase, luminance was kept at 70 cd/m^2^ for two seconds to allow for conscious perception of the ramp aftereffect (Fig. 1D, upper trace). During or latest at the end of the test phase, when the stimulus light was switched off, the participant was expected to press one of three keys indicating their percept. The keys were arranged diagonally across the handheld response device to facilitate blind handling, as fixation was to be maintained at all times. The bottom left key was assigned to a dimming percept, top right key to a brightening percept, and the middle key to a static percept. A keypress immediately ended the test phase. For a lack of ramp aftereffect perception, the run was terminated. If, however, a ramp aftereffect had been reported, the next trial was initiated.

From the second trial onward, all adaptation phases lasted only ten seconds to top up an assumed residual ramp aftereffect. The test phase was also shortened to one second and a counteracting ramping stimulus was displayed (i.e., increasing luminance if a dimming had been reported in the previous test-phase and decreasing luminance after perceived brightening). Hereinafter, the rate of luminance change will be referred to as the “ramp slope.” Using a staircase procedure, the participant further zeroed in on the goal of cancelling out the counter ramp entirely with each trial. Once accomplished, the run was ended through the middle key (perception of steady light). Each condition was presented once following a randomized order. Each such triplet of runs was repeated three times totaling in nine runs. The negative of the counter ramp (symmetry axis at mean luminance) was used as an approximation of ramp aftereffect perception. The steepest counter ramp slopes that were technically achievable were 320 cd/m^2^/s and −70 cd/m^2^/s.

#### Experiment 2: fERG

In the electrophysiological experiment, first and consecutive trials were identical. The adaptation phase followed the same protocol as the psychophysical experiment: presentation of the UpRamp, DownRamp or Control stimulus for 30 seconds (Fig. 1C). After a subsequent 100 ms break, six flashes of 100 ms length followed at a frequency of 2 Hz making the inter stimulus interval 400 ms in length (Fig. 1D, lower trace). Since we expected the continuous light stimulation to keep rods saturated, no background was employed. Flash luminance (150 cd/m^2^) was kept at the lower end of and flash length below the recommended range^16^ to avoid erasure of the ramp aftereffect. Participants were asked to maintain fixation and refrain from blinking during recording. Ten trials intermitted by five-second breaks in darkness were repeated consecutively in one run. Between runs, participants were permitted to take breaks as needed. Three runs were conducted for each condition, with the option to repeat runs (e.g., in cases of excessive blinking or poor data quality). Randomization was the same as in the Nulling experiment (i.e., the three conditions were shown once each in randomized order, which was repeated three times). This resulted in a maximum of 180 possible single responses (six flashes per trial in 30 trials total) per condition before artefact rejection.

### Electrodes and Recording

ERG signals were recorded binocularly with DTL-like electrodes^36^ (Spes Medica S.p.A., Genova, Italy) positioned along the lower eyelid between attachment points at the inner and outer canthus. As reference, a goldcup electrode filled with Medimex conductive gel (Medimex GmbH, Limburg, Germany) was attached to the cleaned (Nuprep skin prep gel; Weaver and Company, Aurora, CO, USA) ipsilateral temple. An ear-clip electrode with conductive gel was used as ground electrode and placed on one cleaned earlobe. Impedance was checked before recording and adjustments made for values above 25 kΩ at all electrodes, with typical values below 5 kΩ for the DTL-like electrodes. Frequencies from 0.5 to 250 Hz were recorded at a sampling rate of 5000 Hz via the BrainRecorder software (BrainVision Recorder 1.10; Brain Products GmbH, Gilching, Germany).

### Data Analysis

**Figure 2.**
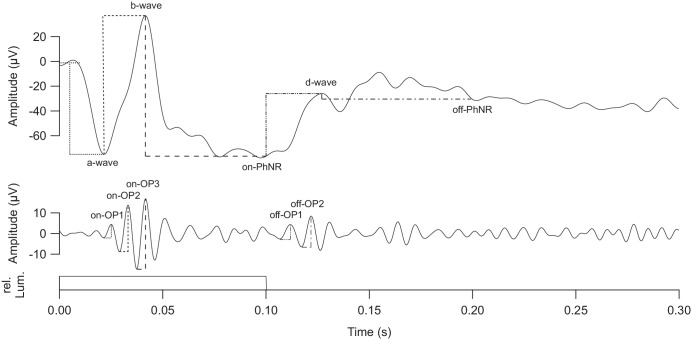
Filtered single ERG response with labeled wave components of interest as voltage over time. *Upper trace*: Frequency band from 0.5 to 80 Hz. The a-wave was measured from baseline to the negative peak. The b-wave was measured from the a-wave trough to the b-wave peak. The on-PhNR was measured from the b-wave peak to the momentary amplitude at light-off (0.1 second). The d-wave was measured from the momentary amplitude at light-off to the d-wave peak. The off-PhNR was measured from the d-wave peak to the mean over a predefined interval (184 to 214 ms). *Middle trace:* Frequency band from 80 to 160 Hz. All OPs were measured relative to the preceding trough. *Bottom trace:* Flash stimulation (100 ms in length) as relative luminance over time.

Analyses, including visualization, was performed with Igor Pro 8 (WaveMetrics Inc., Lake Oswego, OR, USA). A Fourier-transform-based digital notch filter (50 Hz) and artefact rejection (threshold 500 µV) were applied to the data. Signals were cut into segments extending from 0 to 300 ms relative to flash onset, which constitutes the interval of interest, filtered into two frequency bands and averaged across both eyes. One frequency band consisted of signals from 0.5 to 80 Hz and thus showed low-frequency, high-amplitude components like the a-wave, b-wave, d-wave and on- and off-PhNR. The other ranged from 80 to 160 Hz and contained the high-frequency, low-amplitude OPs. Automated peak detection was implemented at a single-trial level, followed by visual inspection. This step also allowed for a general quality check and exclusion of trials that were, for instance, contaminated by obvious artefacts that had not been detected automatically. Per participant an average of 19 trials were rejected from the 180 recorded ones. Amplitudes were computed as a difference between two points in such a way that the normally expected polarity resulted in a positive value. For instance, if an a-wave trough lies below baseline as is typical, its amplitude will be positive. Each component's amplitude was recorded as depicted in Figure 2: a-wave, b-wave and d-wave amplitudes were measured in accordance with the ISCEV standard. The a-wave peak was measured from baseline (0–10 ms) to the first negative peak after light-on, the b-wave from the a-wave trough to the following positive peak and the d-wave as the difference from light-off to the following positive peak.^16^^,^^37^ Because flash length was too short to allow for the on-PhNR to fully reach its potential peak, its amplitude was taken from b-wave peak to light-off as a best approximation. The off-PhNR was highly variable and not always negative-going, hence it was difficult to unequivocally identify its peak. To obtain a reliable approximation, we first determined the PhNR peak in the mean wave across all eyes of all participants. A time interval of 30 ms (184 to 214 ms) around this peak was defined and mapped back to the curves of all individual participants. The average within such an interval was then taken as the individual's PhNR peak level. The difference between this value and the d-wave maximum was taken as the PhNR peak amplitude.

Each OP was measured from the preceding trough to its peak. On-OPs 1, 2 and 3 were defined as the local maxima in the time range between the a-wave trough and the b-wave peak. Occasionally, four peaks fell into that window. Lachapelle et al.^38^ have previously reported a fusion of third and fourth on-OPs into one, when luminance was beneath a certain threshold. Possibly, our parameters were close to this threshold, making the on-OP3 a merge in most cases but occasionally segregated into two peaks. In the latter case the fourth peak entered analysis as it was typically more robust and higher in amplitude than the third peak. Off-OP1 and 2 were defined as the two highest high-frequency peaks around the d-wave peak. Typically, they were superimposed on the ascending d-wave. However, if the d-wave happened to be shifted to very short peak times, the second peak might have occurred after the d-wave's maximum.

All peak times of the on-response were measured from light-on to peak and those of the off-response relative to light-off. The peak times of the on- and off-PhNR were read at fixed times of 0.1 second and 0.1992 second (midpoint of region of interest), respectively.

One-sided paired permutation tests (10,000 repetitions) with subsequent Bonferroni-Holm correction were chosen to test for statistical significance of peak time and amplitude differences between conditions and pairwise between successive flashes 1–6 of the test phases. Statistical dependence was tested with the standard non-parametric Kendall's Tau test for correlations,^39^ followed by Bonferroni-Holm correction.

## Results

### Experiment 1: Nulling

Experiment 

**Figure 3.**
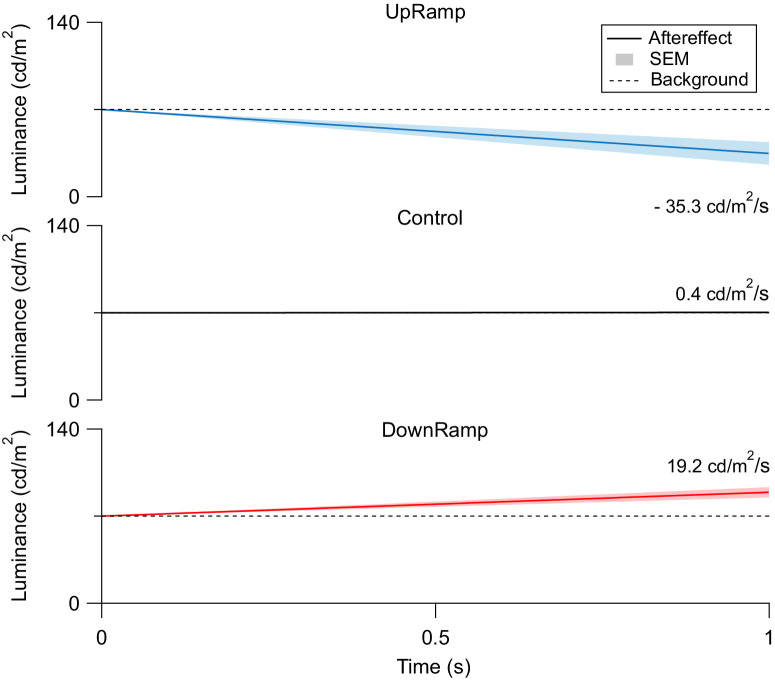
Mean directional strength of the ramp aftereffect. The average ramp aftereffect (*solid line*, *n* = 20) is shown as the subjective change in luminance over time with its standard error of the mean (SEM). In all three conditions, UpRamp (*top*), DownRamp (*bottom*), and Control (*middle*), the ramp aftereffect is superimposed on a background of mean luminance (70 cd/m^2^, *dashed line*). 0 seconds represents the end of adaptation. The mean slopes are given at the right-hand end of each trace. The ramp aftereffect in the sawtooth conditions opposes the ramp of the respective adapting stimulus in direction and is stronger in UpRamp.

The psychophysical experiment used a nulling task to assess ramp aftereffect strength in each of the three adaptation conditions. The negative of the counter ramp slope was used as an indicator of ramp aftereffect strength and directionality. Figure 3 shows this linear approximation of perception for all tested conditions as a mean over all participants. After adaptation with upward ramps, the mean ramp slope of the ramp aftereffect reached −35.3 cd/m^2^/s, a subjective dimming of the light. In the DownRamp condition, the ramp aftereffect was one of brightening at a ramp slope of 19.2 cd/m^2^/s. In both cases, the perceived change in light opposed the ramp phase of the adapting stimulus in its direction, however not with consistent strength. We saw an asymmetry between the conditions, with UpRamp exhibiting the higher absolute ramp slope. The control stimulus barely elicited a ramp aftereffect (slope of 0.4 cd/m^2^/s). On an individual level, these findings were consistent across a large majority of participants.

### Experiment 2: fERG

Experiment 

**Figure 4.**
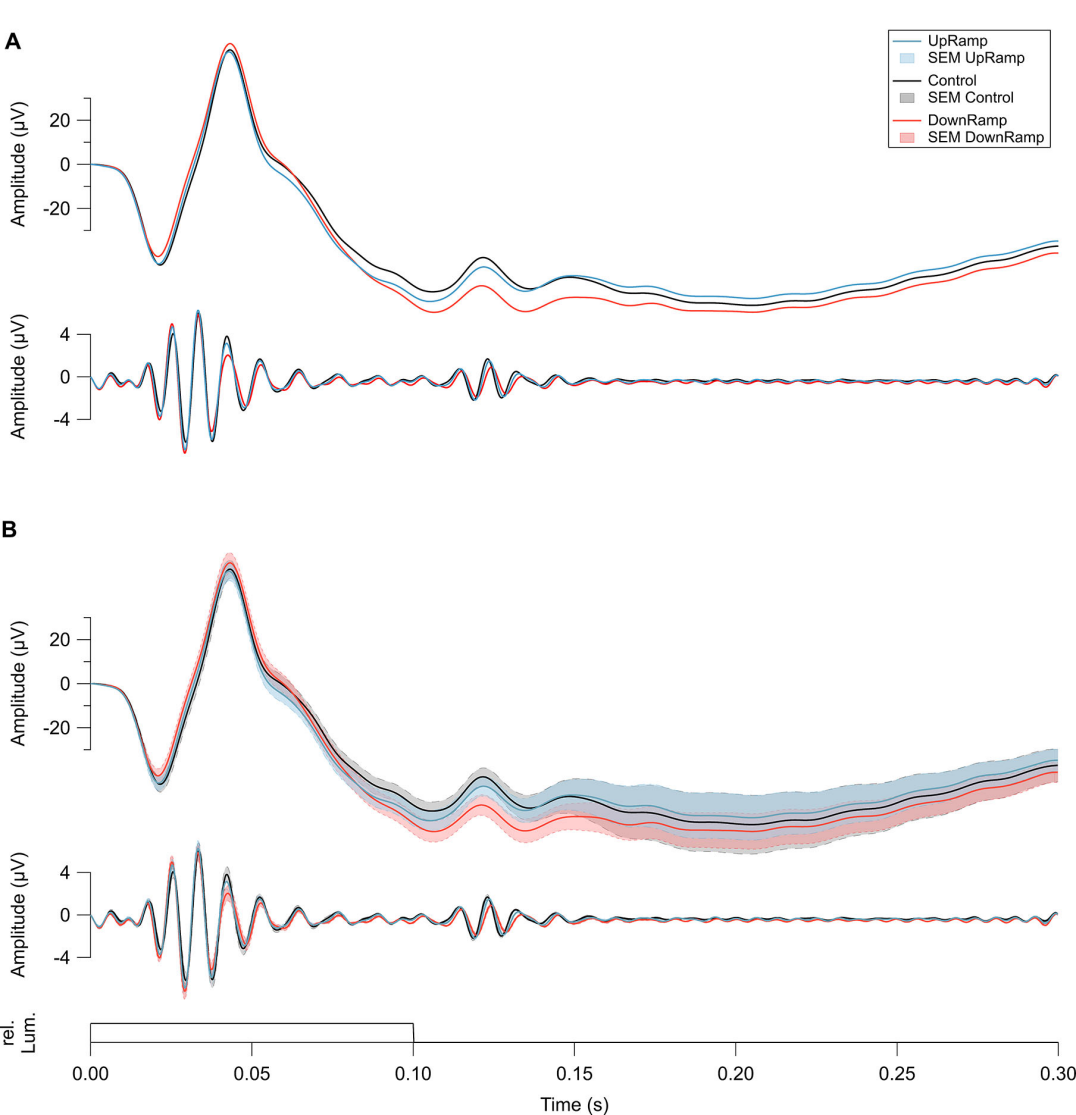
Mean ERG signal for all three conditions in two frequency bands. The same signal is shown in **A** and **B**. In **B**, the grand mean (*n* = 20) of each condition, UpRamp (*blue*), Control (*black*), and DownRamp (*red*), is plotted with its respective standard error of the mean (SEM). The SEM was omitted in **A** to aid comparison of the main traces. In both **A** and **B**, the *upper graph* shows the frequency band of 0.5–80 Hz and the *middle graph* the frequency band of 80–160 Hz, each as voltage change over time. Note the difference in scaling of the ordinate. The *bottom trace* shows the ERG flash (0.1 seconds in length) as relative luminance over time. UpRamp and DownRamp traces can be seen to diverge from Control, as well as from each other.

An exemplary response to a single flash is shown in Figure 2 with every assessed wave component labelled. The grand mean shows both sawtooth conditions to differ from Control, albeit quite subtly (Fig. 4). In particular, the b-wave amplitude in the low-frequency band (0.5–80 Hz, Fig. 4 upper traces in A and B) increased with DownRamp adaptation. Components showing clearly reduced amplitudes in DownRamp are the on-OP3 in the high-frequency band (80–160 Hz, Fig. 4 middle traces in A and B) and the a-wave. Differences in the low frequencies following the b-wave's fall-off are less straightforward to evaluate due to a general divergent signal drift. However, because amplitudes were measured as differences between troughs and peaks, this should not have a major influence on comparisons between conditions. The deviation from Control is not limited to the amplitude, though. In addition, there are changes in response timing. For instance, the a-wave peaks earlier in DownRamp than in Control, whereas the b-wave peak is delayed. Off-OP1 and 2 appear to show clearest the time shift in their delay in DownRamp compared to both UpRamp and Control.

**Figure 5.**
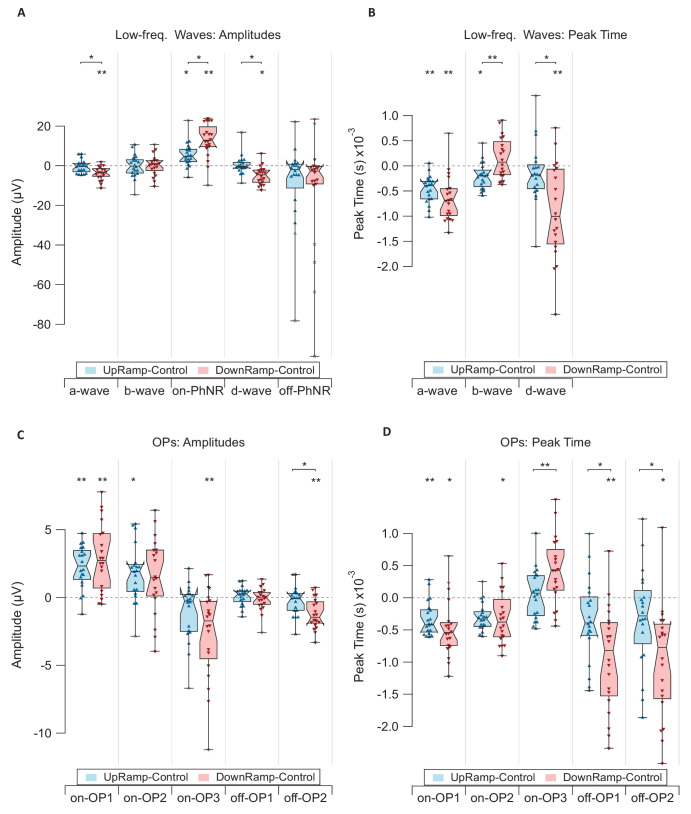
Amplitudes and peak times of all response components in relation to baseline. Specifically, the difference between Control and UpRamp (*blue*) and Control and DownRamp (*red*) is displayed. Each boxplot shows the median (*n* = 20) as its midline and a 95% confidence interval over the length of its notch. Box borders are 25th and 75th percentiles. *Whiskers* span the entirety of datapoints. *Markers* show means per participant. Outliers are drawn as *empty markers* and extreme outliers as *rectangular markers*. Significant differences to Control were marked with *asterisks* above the respective box, and differences between the two sawtooth conditions with *asterisks* at the respective braces. (**A**) Amplitudes of low-frequency waves. (**B**) Peak times of low-frequency waves. (**C**) Amplitudes of oscillatory potentials. (**D**) Peak times of oscillatory potentials. Divergence from Control is mostly uniform in direction for both conditions. Each response component shows significant changes between conditions either in amplitude or peak time.

Timing and amplitudes were analyzed on a single-flash level preventing differences in one dimension to skew measurements in the other after summations and averaging. The boxplots in Figure 5 show measurements in the sawtooth conditions in relation to Control. As peak parameters obtained in the Control condition were subtracted from the respective parameters in each sawtooth condition, positive scores correlate with heightened activity (Figs. 5A and 5C) or delayed peaks (Figs. 5B and 5D). Significance of effects was tested comparing UpRamp with Control, comparing DownRamp with Control, as well as comparing UpRamp with DownRamp using permutation tests (Bonferroni-Holm corrected). The results confirm the changes in amplitudes and peak times already visible in the grand mean and further show significantly altered activity to be present in every investigated response component (Fig. 5). These effects concern timing, amplitude, or both.

The changes were generally more pronounced with DownRamp than with UpRamp. There are few exceptions to this rule in the timing of the b-wave and the amplitudes of on-OP2 and off-OP1. Of these, only the b-wave's peak time reached significant differences between the sawtooth conditions.

With its peak time, the b-wave is also the only response component for which the change in activity was oriented opposingly between conditions. Of the two changes only the delay in UpRamp reached a significant difference to Control, whereas the earlier peaking in DownRamp did not. For all other response components, the changes in amplitude, as well as in timing were identical in their direction.

Besides the b-wave, many other components differed significantly in activity between Up- and DownRamp. In the low-frequency band, that's the a-wave and on-PhNR in their amplitude and the d-wave in both amplitude and peak time. In the high-frequency band, all off-responses and on-OP3 differed significantly in peak time between sawtooth conditions. Off-OP2 additionally varied in amplitude.

Overall, effects were not specific to either light-on or light-off responses. In either case, some components showed an increase and some a decrease in amplitude or peak time. For instance, with Up- and DownRamp both a-wave (on-response) and d-wave (off-response) were reduced in their amplitudes, as well as peak times. The activity of RGCs, though, was captured in two direct equivalents, the on- and off-PhNR, that did show antagonistic changes. Although on-PhNR showed increased amplitudes in both sawtooth conditions, the off-PhNR was reduced in both adaptation conditions. However, the decrease in off-PhNR was not statistically significance in either condition. Nevertheless, the difference between on- and off-PhNR was clearly higher in DownRamp than in UpRamp. It is important to note, however, that both the on- and off-PhNR were in fact only approximations since its peak was not fully reached in case of the on-PhNR or was too broad for precise assessment in case of the off-PhNR.

Although not all assessed differences reached statistical significance, there was an interesting trend emerging in the on-response of OPs during both sawtooth conditions. On-OP1 and on-OP2 both showed an increase in amplitude and a shortened peak time, the opposite, namely reduced amplitude and increased peak time, was seen in on-OP3.

The observed changes diminished rapidly over time, as evident from the evolution of the effect across the six flashes that were shown after each adaptation phase (data not shown). When comparing the amplitude and the peak time of the last probe response to the first probe response for each component and condition, we consistently found a reduction of the effect. The only exception was the on-PhNR amplitude and the a-wave peak time. Even in Control, almost all components were changed in both amplitude and time, with the two aforementioned exceptions plus the on-OP2 timing.

Exploring these effects further, we assessed whether effects of the three adaptation stimuli differ qualitatively between the first and the last probe flash. Hence, the same comparisons between conditions as for the averaged flashes as shown in Figure 5 were repeated for the first and the last flash separately. For the first flash, mostly the same changes were found as for the mean. Except for some minor deviations, the overall picture was again that of many significant differences between conditions spread throughout all neuronal layers of the retina. In contrast, for the last flash, only the decrease of a-wave amplitude in DownRamp compared to Control remained, meaning that the initial effect of adaptation vanished almost entirely over the course of three seconds.

Additionally, there was no sizable correlation across participants between amplitude and peak time of the averaged flashes. Speculating that a higher amplitude might take longer to generate, we tested for such a correlation in all results that had shown a significant effect of adaptation on amplitude or time. There was indeed one wave component (d-wave) that yielded a significant *P* value even after Bonferroni-Holm correction. However, visual inspection did not reveal a clear relationship between amplitude and peak time.

**Figure 6.**
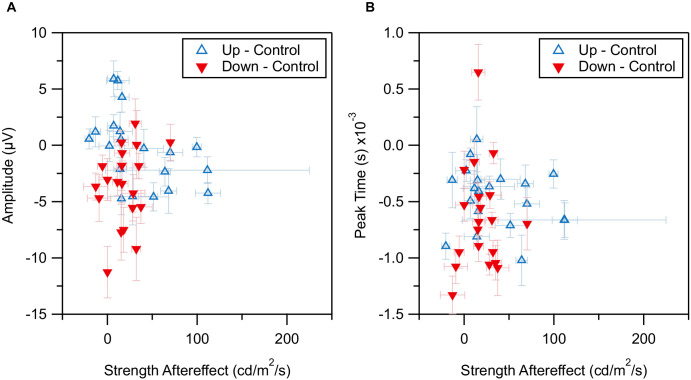
Correlation of electrophysiological and behavioral measures of the ramp aftereffect in the a-wave. Per participant (*n* = 20) mean amplitudes (**A**) or peak time (**B**) of the a-wave were plotted over mean ramp aftereffect strength obtained from the nulling experiment. Each marker represents the mean over all responses from a single participant. All values are differences between Control and one of the two sawtooth conditions, UpRamp (*empty upward pointed triangle*) or DownRamp (*filled downward pointed triangle*). The negative value of the DownRamp aftereffect strength is displayed for easier comparison with the UpRamp data. No correlation is visible between ramp aftereffect strength and amplitude, or peak time.

Furthermore, there was no correlation between retinal activity and subjective perception (Fig. 6). For every component, both amplitude and peak time were tested against the ramp slope of ramp aftereffect perception. None reached significance, irrespective of a correction for multiple testing.

## Discussion

The key findings of the present study are as follows.

• Following adaptation with sawtooth stimuli, significant changes in amplitude and/or peak time were observed in all fERG response components.
• Changes in neuronal activity showed little antagonism between sawtooth adaptation of opposing polarity, although they were generally stronger in DownRamp than UpRamp conditions.
• There was no correlation between perceptual and electrophysiological data.

### Comparison Between UpRamp and DownRamp

The general lack of antagonism between UpRamp and DownRamp conditions in the ERG responses was surprising. The psychophysical assessment revealed the antagonistic sawtooth conditions to induce antagonistic aftereffects in our participants, consistent with past studies.^1^^,^^2^ Hence, we anticipated the neuronal responses to reflect this antagonism between conditions through opposing adaptations. Among the numerous significant differences between UpRamp and DownRamp (Fig. 5), we observed such an opposition exactly once, in the b-wave's peak time (Fig. 5B). Contradicting the selective attenuation of either on- or off-BC activity as proposed by Bosten and MacLeod^3^ (see further discussion below) a distinct shift of peak time in only one type of BCs should consequently affect the b-wave amplitude composed of both on- and off-BC activity.^23^^–^^25^ Such an effect is not found in either condition. Furthermore, the d-wave integrates on- and off-BCs in the off-response.^25^^,^^28^ In the case of attenuated on-BCs in the on-response, we would expect attenuation of off-BCs in the off-response, the d-wave. However, it does not show the same opposition in its adaptive changes. Rather, the response component is changed uniformly across sawtooth conditions as it is in most other components.

The psychophysical experiment—again reproducing past findings^2^—showed stronger changes in perception in UpRamp (Fig. 3). This contrasts with the ERG data, which show stronger overall changes in the Down-Ramp condition (Fig. 5). Because the sawtooth conditions are compared in their difference from baseline, the assessment regarding both the uniformity of direction as well as the strength of adaptation is highly dependent on the control condition.

### Sawtooth Conditions Versus Control

Although the sawtooth stimuli have a directed characteristic (upwards or downwards ramp, complemented by the respective jumps back), retinal mechanisms that adapt to temporal luminance modulation might account for a large part of the differences between sawtooth stimulation and Control. This is reminiscent of the situation with visual motion, where a stimulus moving in a specific direction can result in adaptation of neuronal mechanisms that are sensitive to the flicker that is locally present in a moving stimulus, rather than adaptation of directionally selective mechanisms that would reflect motion proper.^5^ The reason is that a sufficiently small receptive field is stimulated repetitively by alternating luminance levels when exposed to a moving texture, which is identical to being subjected to a flicker stimulus. This assumption is supported by findings that retinal neurons adapt their sensitivity to temporal modulation.^40^^,^^41^ This could possibly be addressed in further studies by using flicker stimuli for adaptation that only have steep flanks (rectangular time course) or consist of alternating upwards and downwards ramps (triangular time course). However, as this only concerns the difference from baseline, direct comparisons between the Up- and DownRamp conditions should be unaffected by adaptation to temporal change.

### The Discrepancy Between Psychophysics and Electrophysiology

The participation of the same individuals and the use of the same adapting stimuli opened the possibility of direct comparisons between psychophysical and electrophysiological data. Both measurements were taken directly after stimulation. However, during nulling the aftereffect was assessed during intervals of one second (with exceptions of first trials: two-second test phases), whereas the series of flashes in the ERG spanned three seconds. A correlation between psychophysical and electrophysiological data would have supported the idea of retinal neuronal adaptation underlying perceptual change. To this end, the difference to the baseline was used for both UpRamp and DownRamp, so that an additional adjustment would result in the same distortion. Still, no correlations were found in any response component (Fig. 6). Carrying the final retinal output, RGCs were the most likely to show any correlation between neuronal activity and perception. The assumption that RGCs encode information about the adaptive state underlying perception is only valid if the effect arises at least partially in the retina. However, additional processing further downstream, for instance in the lateral geniculate nucleus, could make crucial contributions that would not be detected by the ERG. This is on top of a high variance in the PhNR signals, which could not be reliably assessed here.

We acknowledge here that in the psychophysical nulling the equipment did limit the maximally achievable counter ramp for dimming less than for brightening ramp aftereffects. However, three out of the four participants who reached the limit to counteract brightening accepted it as a good match. Because the participants were unaware of possible limits, they could not have prematurely terminated a run because of knowledge of having reached a boundary. The same person who saw an increase in luminance stronger than could be counteracted in some but not all runs was the only person that also reached the limit in countering a dimming effect. The respective participants once accepted the limit as a good final result, and once tried exceeding it. It seems thus that, overall, the technical limitations did not prevent an acceptable measure of ramp aftereffect strength*.*

### Little Evidence of On-/Off-Pathway Involvement

Bosten and MacLeod^3^ proposed that the ramp aftereffect may emerge from reduced sensitivity in the on- (upward ramps) or off-pathway (downward ramps). A comparison between square- and sawtooth-modulated stimuli has shown specifically the response to light increments to be drastically different between squared and ramping waves, while the response to light decrements stayed comparable.^42^ This asymmetry underlines the differential responses of on- and off-pathway to stimulation with sawtooth modulated light. The split into the two pathways occurs at the BCs and influences the shape of the b-wave antagonistically.^25^ If the hypothesis of attenuation was correct, it would be consistent for adaptations to follow to some extent the results of previous chemical blocking studies in primates. Blocking of on-BCs reduces the b-wave's amplitude, whereas blocking of off-BCs enhances it.^25^ We did not find a corresponding pattern in the present data after adaptation (Fig. 5). The opposing effects on b-wave peak time in Up- and DownRamp conditions could stem from distinct attenuation of on- and off-pathways. However, a shift in timing of only on- or only off-BCs (i.e., selectively of the hyperpolarizing or the depolarizing cells) should influence the amplitude of the response superposition that constitutes the b-wave. If adaptation, on the other hand, was caused by a change in both the on- and the off-pathway simultaneously and those changes were opposing in polarity, they might cancel out and leave an unaltered b-wave (e.g., in case of an upregulation in on-BCs and concurrent downregulation in off-BCs in UpRamp). In this example, the higher contribution of faster on-BCs^13^ might decrease the peak time, which is exactly what was measured. In DownRamp, where the opposite regulations would be expected to take place, a corresponding trend but no significant changes from Control were detected. This might be a case of lacking measuring sensitivity in an asymmetric system with differing effect sizes.

The d-wave integrates on- and off-BC activity in the light-off response, which is additionally shaped by photoreceptor activity.^25^^,^^28^ The opposition between conditions seen in the b-wave is not reproduced and no argument can be made here of simultaneous on- and off-pathway adaptation. Measured d-wave amplitudes are partially consistent with blocking studies in that they are significantly reduced with DownRamp adaptation.^29^ This is not the case in the UpRamp condition. Blocking of on-BCs led to larger d-wave amplitudes.^29^ In the DownRamp condition, we found an antagonism between the peak times of the two counterparts, b- and d-wave, potentially hinting at opposing adaptation in the on- and off-pathway. We do not see this relationship with UpRamp adaptation, however.

Interestingly, an antagonistic trend also arises between the PhNR of the on- and off-response, an increase of on-PhNR and a decrease of off-PhNR in both conditions. Because the RGCs forward the retina's final preprocessed signal, any adaptation occurring at preceding processing stages should be reflected by their activity. There was no evidence for this in the present study, although it should be noted that the experimental details were not specifically tailored to assess effects on the PhNR with maximum sensitivity. The ISCEV extended protocol proposes red flashes on a blue background.^43^^,^^44^ Alternatively, RGC activity could be more selectively measured in a pattern ERG.^45^ Because we have found significant retinal changes in this study, further investigation of RGC involvement might be of interest.

### No Clear Effect Propagation

Given the nonspecific but widespread changes over retinal layers, the question of potential effect inheritance along processing pathways arises. Adaptation might form early in the retina with changes being inherited throughout the signaling pathway. However, adaptation to light is known to arise at several stages in the retina, especially the inner retina.^46^ It is thus possible that various effects occur at different processing steps in the retina and collectively change perception to form the ramp aftereffect. The first component of the on-response to show changes in activity is, in fact, the earliest component, the a-wave (Figs. 4, 5). Riddel et al.^47^ have found some evidence in toads that the aftereffect with drifting sawtooth gratings might be caused by photoreceptoral adaptations. Because the a-wave is formed both by photoreceptors^17^^–^^19^ and postreceptoral cells, most likely on-BCs,^20^^–^^22^ it is not possible to determine from an ERG whether the changes occurred distally or in the inner retina. The significance of their amplitude modification is, however, lost in the b-wave without showing even a similar trend of changes. An a-wave change caused by on-BCs could have been visible here, though might not be if balanced out by their push-pull relationship with off-BCs. A change in a-wave potentially caused through photoreceptor activity did not show detectable inheritance to BCs. Postsynaptic to BCs lie RGCs. PhNR amplitudes as estimated in the present study do not demonstrate any relay of significant adaptation effects or a trend of change in the on- or off-pathway, that is from b-wave to on-PhNR and from d-wave to off-PhNR, respectively.

Obviously, interneurons are involved in processing mechanisms and heavily shape information propagation between neurons. Their influence changes the concept of inheritance from a simple series of relay stations to effect propagation through modulation of intraretinal processing. There are indications of amacrine cells, signaling to BCs and RGCs, to contribute to OP formation, most likely on-OP1.^33^ The OPs seem to originate in the inner retina,^33^ that has been shown to greatly contribute to adaptations to light.^46^ In both amplitudes and peak times, significant, as well as nonsignificant, changes are preserved between on-OP1 to on-OP2 and from off-OP1 to off-OP2, as one would expect from an excitatory relationship and from on-OP2 to on-OP3 in an opposing manner, a potential sign-reversing connection. These are obviously highly speculative because their synaptic connectivity is still not clearly understood.

## Conclusions

Although we found extensive evidence of adaptation in the retina, the present findings do not support the notion of such adaptation being specific to the ramp direction. The data certainly do not completely rule out a contribution of retinal mechanisms to the generation of the perceptual ramp aftereffect, with a lack of ERG correlates possibly resulting from insufficient signal-to-noise ratio or cancellation between pathways. Primarily, however, the present results prompt for the investigation of post-retinal factors.
